# Supplementary material for: Conditional chemoconnectomics (cCCTomics) as a strategy for efficient and conditional targeting of chemical transmission
Source: eLife. 2024 Apr 30;12:RP91927. doi: 10.7554/eLife.91927 (PMC11060718; doi:10.7554/eLife.91927)
Supplement: Figure 3—source data 3. [file elife-91927-fig3-data3.zip › Figure 3 source data3 part 1.zip!/Figure 3-Source data 3-part1/1063-13-CRISPResso_Report_8cz_fsdr.zip!/CRISPResso_on_1063_14/9.Alleles_frequency_table_around_sgRNA_cgtcgagcacatgacatcaa.pdf]

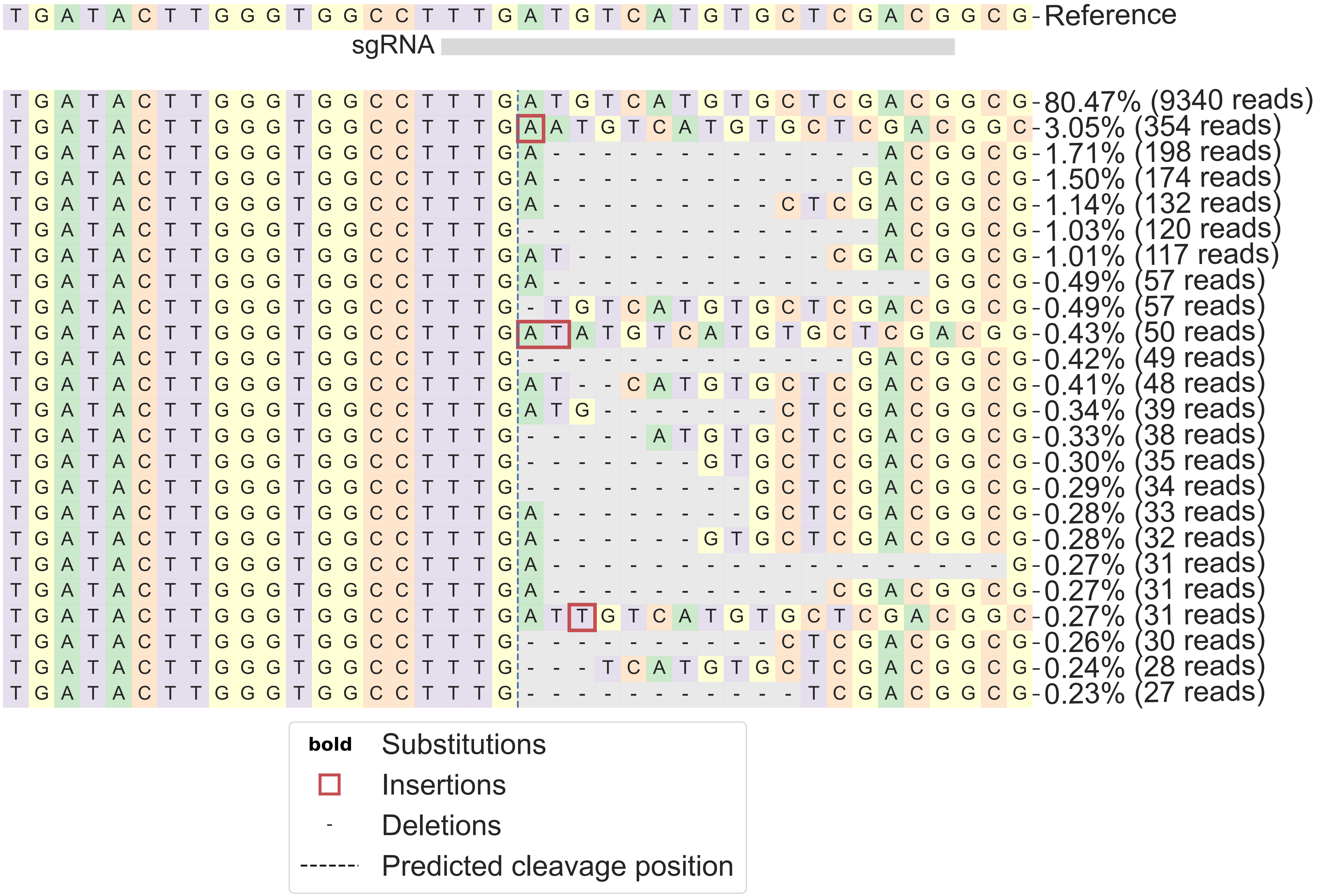

TGATACTTGGGTGGCCTTTGATGTCATGTGCTCGACGGCG - Reference
sgRNA
TGATACTTGGGTGGCCTTTGATGTCATGTGCTCGACGGCG - 80.47% (9340 reads)
TGATACTTGGGTGGCCTTTGAATGTCATGTGCTCGACGGC - 3.05% (354 reads)
TGATACTTGGGTGGCCTTTGA-------------ACGGCG - 1.71% (198 reads)
TGATACTTGGGTGGCCTTTGA------------GACGGCG - 1.50% (174 reads)
TGATACTTGGGTGGCCTTTGA---------CTCGACGGCG - 1.14% (132 reads)
TGATACTTGGGTGGCCTTTG--------------ACGGCG - 1.03% (120 reads)
TGATACTTGGGTGGCCTTTGAT----------CGACGGCG - 1.01% (117 reads)
TGATACTTGGGTGGCCTTTGA---------------GGCG - 0.49% (57 reads)
TGATACTTGGGTGGCCTTTG-TGTCATGTGCTCGACGGCG - 0.49% (57 reads)
TGATACTTGGGTGGCCTTTGATATGTCATGTGCTCGACGG - 0.43% (50 reads)
TGATACTTGGGTGGCCTTTG-------------GACGGCG - 0.42% (49 reads)
TGATACTTGGGTGGCCTTTGAT--CATGTGCTCGACGGCG - 0.41% (48 reads)
TGATACTTGGGTGGCCTTTGATG-------CTCGACGGCG - 0.34% (39 reads)
TGATACTTGGGTGGCCTTTG-----ATGTGCTCGACGGCG - 0.33% (38 reads)
TGATACTTGGGTGGCCTTTG-------GTGCTCGACGGCG - 0.30% (35 reads)
TGATACTTGGGTGGCCTTTG---------GCTCGACGGCG - 0.29% (34 reads)
TGATACTTGGGTGGCCTTTGA--------GCTCGACGGCG - 0.28% (33 reads)
TGATACTTGGGTGGCCTTTGA------GTGCTCGACGGCG - 0.28% (32 reads)
TGATACTTGGGTGGCCTTTGA------------------G - 0.27% (31 reads)
TGATACTTGGGTGGCCTTTGA-----------CGACGGCG - 0.27% (31 reads)
TGATACTTGGGTGGCCTTTGATTGTCATGTGCTCGACGGC - 0.27% (31 reads)
TGATACTTGGGTGGCCTTTG----------CTCGACGGCG - 0.26% (30 reads)
TGATACTTGGGTGGCCTTTG---TCATGTGCTCGACGGCG - 0.24% (28 reads)
TGATACTTGGGTGGCCTTTG-----------TCGACGGCG - 0.23% (27 reads)
bold Substitutions
Insertions
- Deletions
Predicted cleavage position
